# L-serine supplementation lowers diabetes incidence and improves blood glucose homeostasis in NOD mice

**DOI:** 10.1371/journal.pone.0194414

**Published:** 2018-03-15

**Authors:** Laurits J. Holm, Martin Haupt-Jorgensen, Jesper Larsen, Jano D. Giacobini, Mesut Bilgin, Karsten Buschard

**Affiliations:** 1 The Bartholin Institute, Department of Pathology, Rigshospitalet, Copenhagen, Denmark; 2 Cell Death and Metabolism Unit, Center for Autophagy, Recycling and Disease, Danish Cancer Society Research Center, Copenhagen, Denmark; Children's Hospital Boston, UNITED STATES

## Abstract

Sphingolipids are a diverse group of lipids with important roles in beta-cell biology regulating insulin folding and controlling apoptosis. Sphingolipid biosynthesis begins with the condensation of L-serine and palmitoyl-CoA. Here we tested the effect of L-serine supplementation on autoimmune diabetes development and blood glucose homeostasis in female NOD mice. We found that continuous supplementation of L-serine reduces diabetes incidence and insulitis score. In addition, L-serine treated mice had an improved glucose tolerance test, reduced HOMA-IR, and reduced blood glucose levels. L-serine led to a small reduction in body weight accompanied by reduced food and water intake. L-serine had no effect on pancreatic sphingolipids as measured by mass spectrometry. The data thus suggests that L-serine could be used as a therapeutic supplement in the treatment of Type 1 Diabetes and to improve blood glucose homeostasis.

## Introduction

Type 1 Diabetes is an autoimmune disease in which the insulin producing beta cells are either destroyed by autoreactive T cells or dysfunctional leading to insulin deficiency and hyperglycemia [1]. The development of Type 1 Diabetes is influenced by several factors, including genetic susceptibility [2], and environmental factors such as diet, viruses, and microorganism [3]. The incidence of Type 1 Diabetes is increasing worldwide, highlighting the need for effective and affordable prevention strategies [4, 5], with current therapeutic approaches focusing on islet transplantation and immune modulation [1].

The NOD mouse is the preferred animal model for studying autoimmune diabetes, as it develops diabetes spontaneously with similarities to human Type 1 Diabetes [6]. Numerous treatments are known to prevent or reverse autoimmune diabetes in NOD mice, however these have not been successful in human trials [7].

Growing evidence suggest a key role for sphingolipids in the pathogenesis of Type 1 Diabetes [8–10]. Sphingolipids, a heterogeneous class of lipids defined by their sphingoid backbone, are formed by serine palmitoyltransferase in a condensation reaction of L-serine and palmitoyl-CoA [11, 12]. Various sphingolipids have important roles in beta-cell biology, regulating insulin folding and secretion, proliferation, apoptosis, and the immune system [13–16]. The sphingolipid precursor L-serine can be made *de novo* through the phosphorylated pathway and is as such classified as a non-essential amino acid, however an external supply of L-serine is necessary for the required level of sphingolipid synthesis in the central nervous system [17, 18]. Dietary supplementation of L-serine is known to increase the amount of plasma sphingolipids in both mice and humans [19]. Highly interestingly, both Type 2 Diabetes patients and patients with gestational diabetes have reduced levels of L-serine in serum [20, 21], thus suggesting that a lack of L-serine is a general condition across different types of diabetes.

Here we tested the hypothesis that L-serine supplementation could prevent autoimmune diabetes and improve blood glucose homeostasis in NOD mice by modulating pancreas sphingolipid composition.

## Materials and methods

### Animals, diet, and water intake

All animal experiments were conducted with permission from the Danish Animal Experiments Inspectorate (reference 2016-15-0201-00841) and the local ethical committee (EMED: P 16–243). Experiments was performed according to international guidelines for the care and use of laboratory animals. Female NOD mice (Taconic Biosciences, Germantown, NY, USA) were housed at the Department of Experimental Medicine, Biocenter, University of Copenhagen, Denmark and kept in a specific Pathogen-Free (SPF) animal facility (temperature 22°C, 12 h light cycle, air change 16 times per hour, and humidity 55± 10%). Three-week-old mice were acclimatized for one week with free access to standard chow diet Altromin 1320 (Altromin, Lage, Germany) and water. Age and weight matched mice were divided in two groups, both with free access to Altromin 1320 (Altromin) and to water with or without the addition of L-serine (AppliChem, Darmstadt, Germany) 85.7g/L, or approximately 280mg/day/mouse. L-serine supplemented water was pH adjusted. Mice were treated from the age of four weeks and until the experiment was terminated at age 45 weeks. Mice were weighed each week and food and water intake were measured weekly by weighing of the food racks or water flask, respectively. The chow diet contained 3188kcal/kg, L-serine supplemented water was set to contain 342.8kcal/L. Sample sizes were based on previous studies with similar experimental settings [22].

### Diabetes monitoring

Mice were inspected weekly for autoimmune diabetes using FreeStyle Lite (Abbott Diabetes Care, Alameda, CA, USA) glucose monitoring. Diagnosis was based on two tail blood glucose measurements with an interval of two days ≥12mM. Blood glucose was measured between 9am and 12pm. Diabetic animals were sacrificed by cervical dislocation.

### Insulitis scoring

Pancreata from 13-week-old mice were dissected and fixed in 10% neutral buffered formalin overnight and embedded in paraffin. 5μm sections were cut and subsequently stained with haematoxylin and eosin. Sections were evaluated in a blinded fashion using an BX53 microscope (Olympus America, Inc., Melville, NY, USA). 30 islets from each mouse were scored according to the following scale: 0, no infiltration; 1, intact islets but with few mononuclear cells surrounding the islets; 2, peri-insulitis (multiple mononuclear cells surrounding the islets); 3, islet infiltration below 50%; 4, islet infiltration above 50%. See S1 Fig for images corresponding to the different insulitis levels.

### Homeostatic model assessment

For calculation of homeostasis model assessment of insulin resistance (HOMA-IR) and beta-cell function (HOMA-Beta), the mice were fasted for six hours and blood glucose measured as described above. Mice were killed by cervical dislocation and blood was collected immediately by heart puncture. Insulin concentration was measured in blood serum using Mercodia Mouse Insulin ELISA kit (Mercodia, Uppsala, Sweden). HOMA-IR was calculated as $\frac{fasting\;serum\;insulin\;content\;mU/L\;\times \;fasting\;blood\;glucose\;mM}{22.5}$ and HOMA-Beta as $\frac{20\;\times \;fasting\;serum\;insulin\;content\;mU/L}{fasting\;blood\;glucose\;mM-3.5}$.

### Glucose tolerance test (GTT)

Mice were fasted 6h and intraperitoneally injected with 0.01ml 1M glucose/g body weight. Blood was drawn from the tail and glucose concentrations were measured as described above at time 30, 45 60, 90, and 120 minutes. Incremental area under the curve (AUC) was calculated using the fasting blood glucose level prior to glucose administration as baseline.

### Lipid measurement

For lipid measurement 10mg samples were taken from the tale of the pancreas, snap frozen and kept at -80°C until analysis. Samples were homogenized at 4°C on TissueLyser II (QIAGEN, Hilden, Germany) in 155mM ammonium acetate. Total protein concentration was measured using Pierce BCA Protein Assay (Thermo Fisher Scientific, Waltham, MA, USA).

Aliquots corresponding to 100μg protein were subjected to lipid extraction by a modified Bligh and Dyer protocol executed at room temperature [23]. The sample aliquots were spiked with 10μL of 50nM, corresponding to 0.5pmol of SHexCer 30:1:2 standard. Quantification of sulfatide species were performed on a (U)HPLC UltiMate 3000 RSLCnano System (Thermo Fisher Scientific) interfaced on-line to quadrupole-orbitrap mass spectrometer Q-Exactive (Thermo Fisher Scientific). We used a silica column 0.5 x 150mm (YMC-Pack Silica analytical column, 3μm particles). We used Lipid Xplorer (https://wiki.mpi-cbg.de/lipidx/Main_Page) to extract data, selecting a time range based on the eluted peaks of the sulfatide species. See S2 Fig for the structure of sulfatide and the identification of the peak and retention time. For the quantitative shotgun lipidomics analysis, aliquots corresponding to 20μg of protein were subjected to lipid extraction by a modified two-step Bligh and Dyer protocol executed on ice [24, 25]. The aliquots of tissue homogenate was spiked with 15μL internal lipid standard mix containing 30pmol cholesteryl ester 15:0-D7, 20pmol ceramide 18:1;2/12:0;0, 10pmol diacylglycerol 12:0/12:0, 20pmol dihexose ceramide 18:1;2/12:0;0, 25pmol hexose ceramide 18:1;2/12:0;0, 25pmol lysophosphatic acid 17:0, 20 pmol lysophosphatidylcholine 10:0, 25pmol lysophosphatidylethanolamine 13:0, 15pmol lysophosphatidylglycerol 17:1, 20pmol lysophosphatidylinositol 13:0, 20pmol lysophosphatidylserine 17:1, 25pmol phosphatidic acid 12:0/12:0, 20pmol phosphatidyl choline ether 18:1/18:1, 25pmol phosphatidyl ethanolamine 12:0/12:0, 15pmol phosphatidylglycerol 12:0/12:0, 15pmol phosphatidylinositol 8:0/8:0, 20pmol phosphatidylserine 12:0/12:0, 20pmol, and 20pmol sphingomyeline 18:1;2/12:0;0. The combined two-step lipid extracts were subjected to mass spectrometric analysis using a quadrupole-orbitrap mass spectrometer Q-Exactive equipped with a TriVersa NanoMate (Advion Biosciences, Ithaca, NY, USA). Data processing was preformed using LipidX. All solvents were HPLC-grade for lipid extraction and for LC-MS analysis all solvents were LC-MS grade. Methanol, water, and chloroform for lipid extraction were supplied by Rathburn (Walkerburn, Scotland). Water, methanol, and acetonitrile for LC-MS were supplied by VWR (Radnor, PA, USA). Ammonium acetate was supplied by Sigma Aldrich (Darmstadt, Germany).

### Statistics

Mixed model analysis of blood glucose levels and weight was performed using SAS Enterprise v7.11 (SAS institute Inc, NC, USA). Final model was adjusted for time, there were no time-diet interaction. The remaining statistical analysis was performed using Graphpad Prism version 6.01 (La Jolla, CA, USA) and data is shown as mean ±SEM unless otherwise noted. The cumulative diabetes incidence was assessed using Mantel-Cox log-rank test. For comparisons between groups, a two-tailed unpaired Student’s *t*-test was used. For multiple comparisons between two groups a two-tailed unpaired Student’s *t*-test, corrected for multiple comparisons using the Holm-Sidak method was used. A p-value of less than 0.05 was considered significant. *p<0.05; **p<0.01; ***p<0.001.

## Results

### Reduced diabetes incidence and insulitis in L-serine supplemented mice

**Fig 1:**
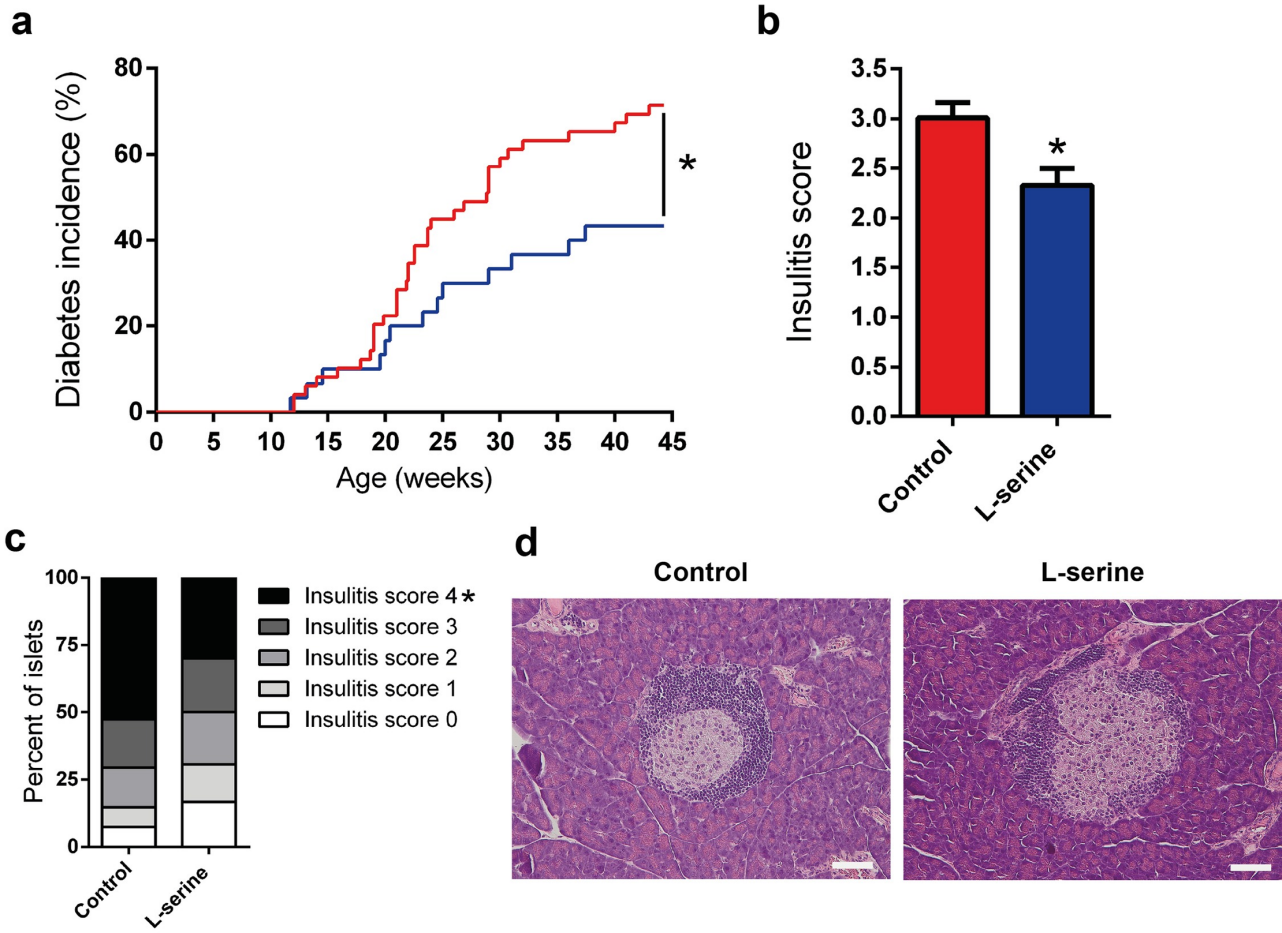
L-serine reduces autoimmune diabetes incidence and insulitis score in NOD mice. (a) Kaplan-Meier survival curves showing diabetes incidence up to 45 weeks of age. Blood glucose was measured once a week and diabetes diagnosis were based on two blood glucose measurements with an interval of two days ≥12mM, control (n = 49) and L-serine (n = 30), p = 0.02. (b) Insulitis score in 13-weeks-old NOD mice, 30 islets were scored per mouse. n = 5. Shown is mean ± SEM. p = 0.02. (c) Percentage distribution of insulitis levels: 0 (white), 1 (light grey), 2 (grey), 3 (dark grey), and 4 (black). p = 0.03, for insulitis level 4. (d) Representative images of insulitis level in control and L-serine mice. Scale bar, 50 μm. Control is shown in red and L-serine in blue. Statistical tests: Mantel-Cox log-rank test (a) and unpaired two-tailed Student’s *t*-test (b, c). *p<0.05.

To study the therapeutic potential of L-serine, NOD mice were treated with water containing L-serine (85.7g/L) from age four weeks onwards. Treatment with L-serine significantly lowered the incidence of autoimmune diabetes to 43% (13/30) compared to controls 71% (35/49) (Fig 1A). Next, we correlated this to the immunological activity in pancreas by looking at islet infiltration. Insulitis score was reduced in the L-serine supplemented group compared to control (Fig 1B). This was clearly seen from islets with insulitis score 4, which was 30% in the L-serine treated mice and 52.6% in controls (Fig 1C).

### L-serine improves GTT, reduces HOMA-IR, and blood glucose

**Fig 2:**
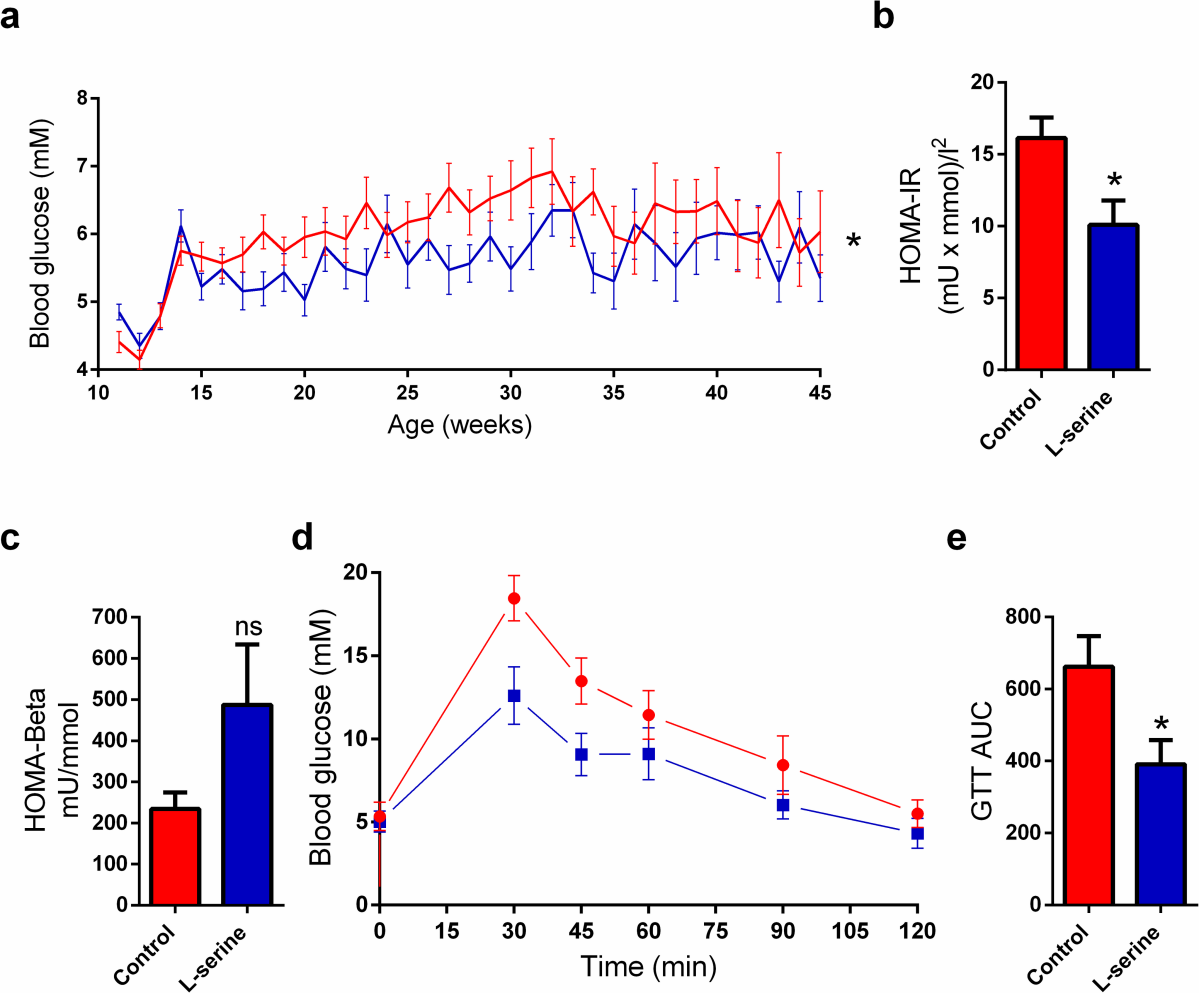
L-serine supplementation improves glucose homeostasis. (a) Blood glucose levels in healthy NOD mice up to 45 weeks of age, control (n = 46) and L-serine (n = 30), p = 0.047. Homeostasis model assessment of insulin resistance (HOMA-IR) (a) and beta-cell function (HOMA-Beta) (b) was assessed in mice age 13 weeks based on fasting insulin and blood glucose concentration (n = 4) p = 0.03 and p = 0.15, respectively. (d) Glucose tolerance test (GTT) was performed on healthy NOD mice age 45 weeks, which were injected with 1M glucose 0.01ml/g body weight. Blood glucose was measured at the indicated time (n = 5). (e) Area under the curve (AUC) calculation for the GTT, p = 0.04. Control is shown in red and L-serine in blue. Statistical tests: mixed model (a) and unpaired two-tailed Student’s *t*-test (b, c, and e). Data shown as mean ± SEM. *p<0.05.

Next, we wanted to assess the effect of L-serine on blood glucose homeostasis. We found, based on weekly blood glucose measurements, that the L-serine treated mice throughout the treatment period had a lower average blood glucose than controls (5.5mM vs 5.9mM) (Fig 2A). HOMA-IR and HOMA-Beta is a mathematical method used to quantify insulin resistance and beta-cell function based on fasting insulin and glucose levels. We measured fasting blood glucose and serum insulin levels in 13-week-old mice and found that L-serine decreased HOMA-IR (Fig 2B), and trended towards better beta-cell function, as evidenced by increased HOMA-Beta (Fig 2C). At the end of the experiment, mice aged 45 weeks were examined for their ability to clear glucose with a GTT. The GTT showed that L-serine treated mice had an improved glucose tolerance (Fig 2D and 2E).

### L-serine supplementation reduces body weight

**Fig 3:**
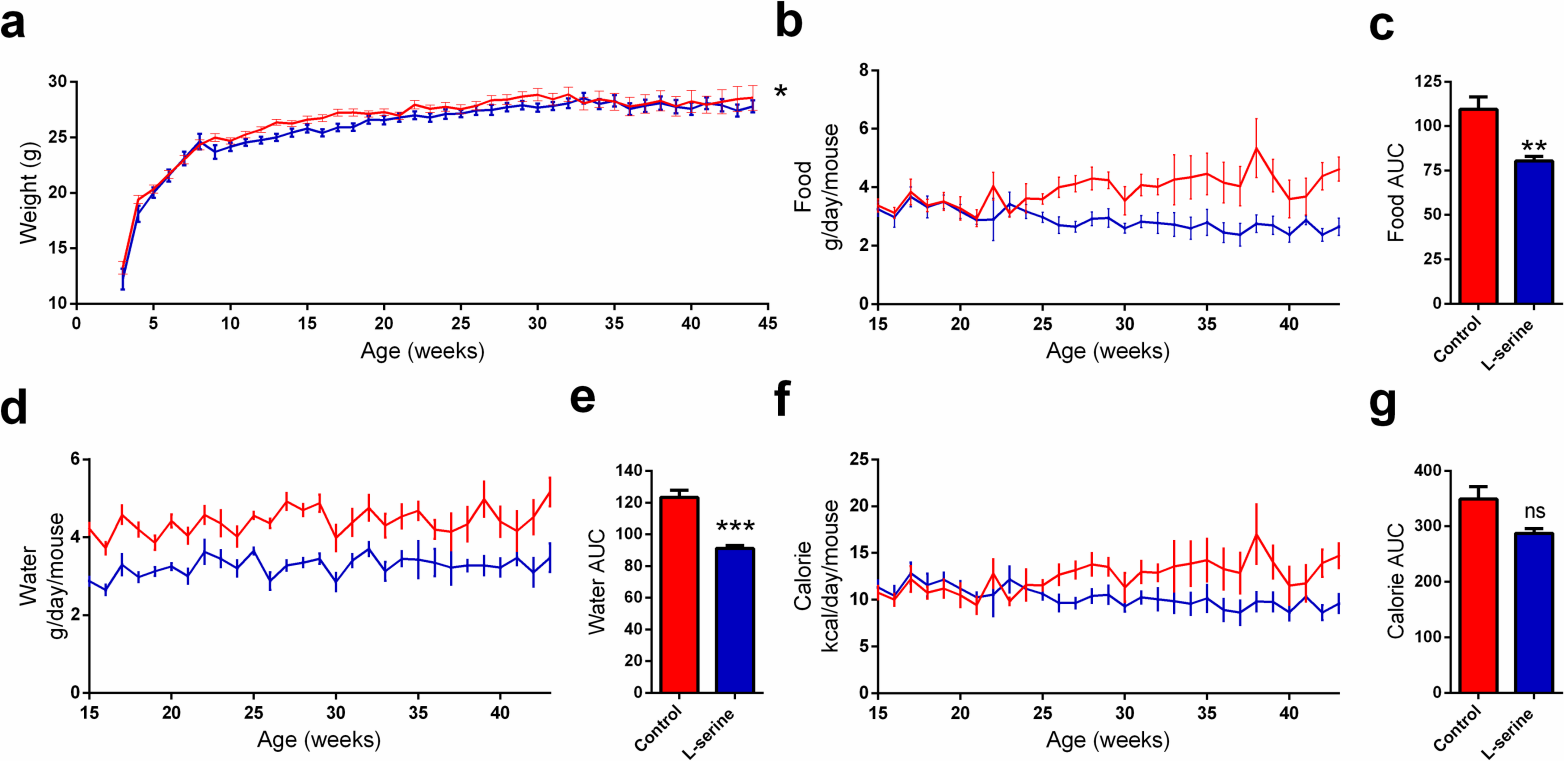
L-serine supplementation reduces body weight. (a) Body weight as measured once a week in healthy NOD mice, control (n = 49), and L-serine (n = 30). p = 0.045 (b) Food intake was calculated per cage by weighing the food racks, control (n = 5) and L-serine (n = 4). (c) Area under the curve (AUC) calculation for food intake, p = 0.01. d) Water intake as calculated per cage by weighting of water flasks, control (n = 5) and L-serine (n = 4). AUC calculation for water intake, p = 0.001. (f) Calorie intake was calculated to adjust for the intake of L-serine through water, control (n = 5) and L-serine (n = 4). AUC calculation for calorie intake, p = 0.054. Control is shown in red and L-serine in blue. Statistical tests: mixed model (a) and unpaired two-tailed Student’s t-test (c, e g). Data is shown as mean ± SEM. *p<0.05; **p<0.01; ***p<0.001.

Weight and calorie intake are known to influence blood glucose homeostasis and so we wanted to evaluate if L-serine had any effect on body weight. The initial weight gain in the two groups were similar; however, at around eight-weeks of age the weight gain was lower in the L-serine treated mice. The difference in body weight was maintained for the next 25 weeks (Fig 3A). L-serine treated mice weighted on average 0.5g less than controls throughout the treatment period. This was associated with a reduced food and water intake in the L-serine group (Fig 3B–3E). This significant difference was however aborted when adjusting for the extra calories consumed through the L-serine supplemented water (Fig 3F and 3G).

### L-serine supplementation does not change pancreatic sphingolipid content

Next, we wanted to evaluate the effect of L-serine supplementation on the composition of sphingolipid in pancreas. 10μg samples from the pancreatic tail were taken from mice age 45 weeks and analysed by mass spectrometry. Five sphingolipid species were identified in the pancreas (ceramide, hexose ceramide, dihexose ceramide, sphingomyeline, and sulfatide). Of these sulfatide, an insulin chaperone [15], trended towards reduced levels following L-serine treatment (Table 1). An additional 20 lipid species were identified in the pancreas with the large majority consisting of phosphatidyl choline. However, we find no differences between the groups (for data on all lipids see S1 Table).

**Table 1:** Pancreas lipid content.

Lipid	Control (SD)	L-serine (SD)	p value
Cholesteryl ester	0.59 (0.57)	0.73 (1.13)	>0.99
Ceramide	62.37 (13.45)	67.79 (14.59)	>0.99
Diacylglycerol	12.86 (15.88)	11.94 (11.51)	>0.99
Dihexose ceramide	0.05 (0.07)	0.11 (0.17)	>0.99
Hexose ceramide	0.05 (0.07)	0.14 (0.09)	0.92
Lysophosphatic acid	43.01 (17.81)	37.74 (13.59)	>0.99
Lysophosphatidylcholine	232.01 (8.28)	215.22 (89.09)	>0.99
Lysophosphatidylcholine ether	0.44 (0.18)	0.44 (0.28)	>0.99
Lysophosphatidylethanolamine	105.28 (12.67)	129.34 (25.96)	0.9
Lysophosphatidylethanolamine ether	9.38 (5.7)	14.93 (7.65)	0.99
Lysophosphatidylglycerol	0.84 (0.31)	2.36 (2.49)	0.99
Lysophosphatidylglycerol ether	0.10 (0.06)	1.31 (1.68)	0.96
Lysophosphatidylinositol	18.82 (10.97)	22.34 (13.32)	>0.99
Lysophosphatidylinositol ether	0.20 (0.17)	0.27 (0.23)	>0.99
Lysophosphatidylserine	27.57 (16.14)	42.97 (19.58)	0.99
Phosphatidic acid	1.65 (1.49)	0.95 (0.72)	>0.99
Phosphatidyl choline	3869.27 (162.62)	3805.88 (81.13)	>0.99
Phosphatidyl choline ether	50.42 (4.66)	38.61 (5.93)	0.18
Phosphatidyl ethanolamine	160.23 (32.82)	186.20 (44.62)	>0.99
Phosphatidyl ethanolamine ether	83.95 (26.95)	80.92 (9.62)	>0.99
Phosphatidylglycerol	3.89 (1.33)	3.67 (1.21)	>0.99
Phosphatidylinositol	87.07 (9.23)	102.84 (7.02)	0.31
Phosphatidylserine	19.48 (1.38)	20.39 (3.68)	>0.99
Sulfatide	1.81 (0.37)	0.94 (0.39)	0.16
Sphingomyeline	210.46 (44.71)	212.91 (24.69)	>0.99

Lipid content in pancreas. 10μg tissue samples were analysed by mass spectrometry. Lipid amount is shown as pmol/mg protein. Data is mean ± SD. Statistical tests: two-tailed unpaired Student’s *t*-test, corrected for multiple comparisons using the Holm-Sidak method.

## Discussion

In the current study, we tested the hypothesis that dietary supplementation of the sphingolipid precursor L-serine would be protective against autoimmune diabetes in NOD mice by regulating pancreas sphingolipid composition. We found that L-serine reduced diabetes incidence and reduced inflammation in pancreatic islets (Fig 1A–1C). Furthermore, we found that L-serine improved glucose homeostasis as evidenced by improved glucose tolerance, reduced HOMA-IR, and reduced average blood glucose (Fig 2A–2D). High protein diets are otherwise normally associated with increased risk of insulin resistance and Type 2 Diabetes [26]. However, our study indicate that this effect might be dependent on the amino acid composition. Similar positive effects have been described for L-leucine [27]. Weight and calorie intake are well known modulators of insulin resistance [28], and we found that L-serine treatment resulted in a small reduction in body weight, which could be explained by the trend towards lower calorie intake. (Fig 3A, f-g). Hence, we cannot rule out that the changes in body weight and food intake can be part of the explanation of the observed results. Together these results suggest that supplementation of L-serine could have beneficial effects regarding prevention of Type 1 Diabetes. Sphingolipids are important regulators of beta-cell survival and function with different sphingolipid species having opposite effects. Ceramide inhibits beta-cell proliferation and insulin expression while also inducing apoptosis [29]. Sulfatide on the other hand promotes insulin folding and preserves insulin crystals [15, 30]. We therefore hypothesized that the beneficial effect of L-serine would be reflected in pancreas sphingolipids composition. However, we observed no significant changes in pancreas sphingolipids and surprisingly even a trend towards lower amounts of sulfatide (Table 1). Administration of sulfatide have otherwise previously been shown to reduced diabetes incidence in NOD mice to a similar degree as L-serine [31]. Sphingolipids are regulators of lipid metabolism [32] and in similarity to sphingolipids then we observe no changes in the pancreatic lipidome. It should be noted that we performed lipid analysis on whole pancreas and thus islet specific changes might not detectable in our study. The mechanism behind the beneficial effect of L-serine described here remains unidentified. An explanation, might rely on the insulinotropic properties of L-serine [33] and so increased insulin secretion could improve postprandial glucose levels. The observed reduction in blood glucose levels suggest reduced beta-cell stress, which have been linked to the initiation of insulitis [34]. In conclusion, L-serine supplementation could be used as a therapeutic supplement for prevention of Type 1 Diabetes as show by its beneficial effect on diabetes development, degree of insulitis, and positive effects on blood glucose homeostasis in NOD mice.

## Supporting information

S1 Fig Insulitis levels. Shown are representative examples of the different insulitis scores as seen in NOD mice age 13 weeks. Scale bar, 50 μm.
(TIF)

S2 Fig Identification of sulfatide species. (a) Structure, molecular formula, and monoisotopic mass of sulfatide 30:1;2, which was used as an internal standard for the mass spectrometry analysis. (b) Negative ionization of the standard SHexCer 30:1;2. Below, further identification of the peak and retention time corresponding to its m/z = 722.4519.
(TIF)

S1 Table Pancreatic lipid content. List of all identified lipids and amount found in the pancreas of the examined mice. Lipid amount is shown as pmol/mg protein.
(XLSX)
