# Supplementary figures and images for: L-serine supplementation lowers diabetes incidence and improves blood glucose homeostasis in NOD mice
Source: PLoS One. 2018 Mar 15;13(3):e0194414. doi: 10.1371/journal.pone.0194414 (PMC5854405; doi:10.1371/journal.pone.0194414)

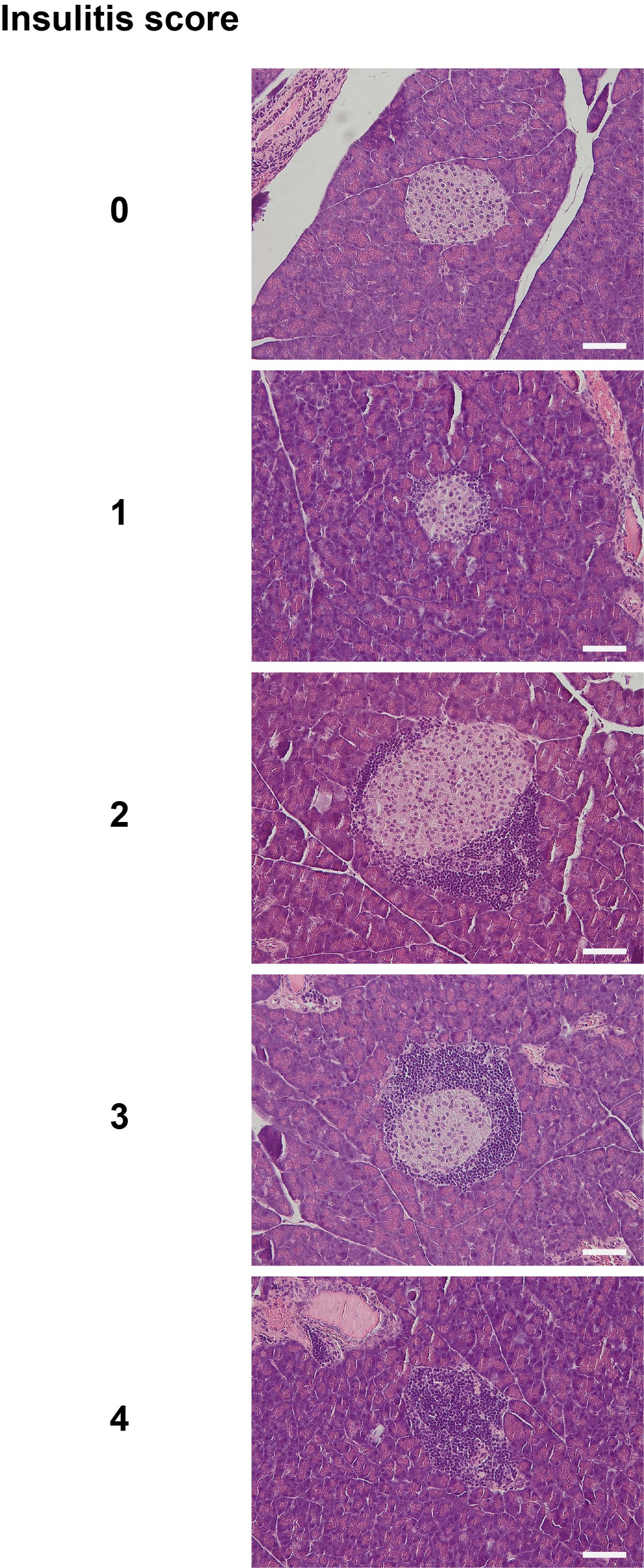

Supplement: S1 Fig — Shown are representative examples of the different insulitis scores as seen in NOD mice age 13 weeks. Scale bar, 50 μm. (TIF) [file pone.0194414.s001.tif]

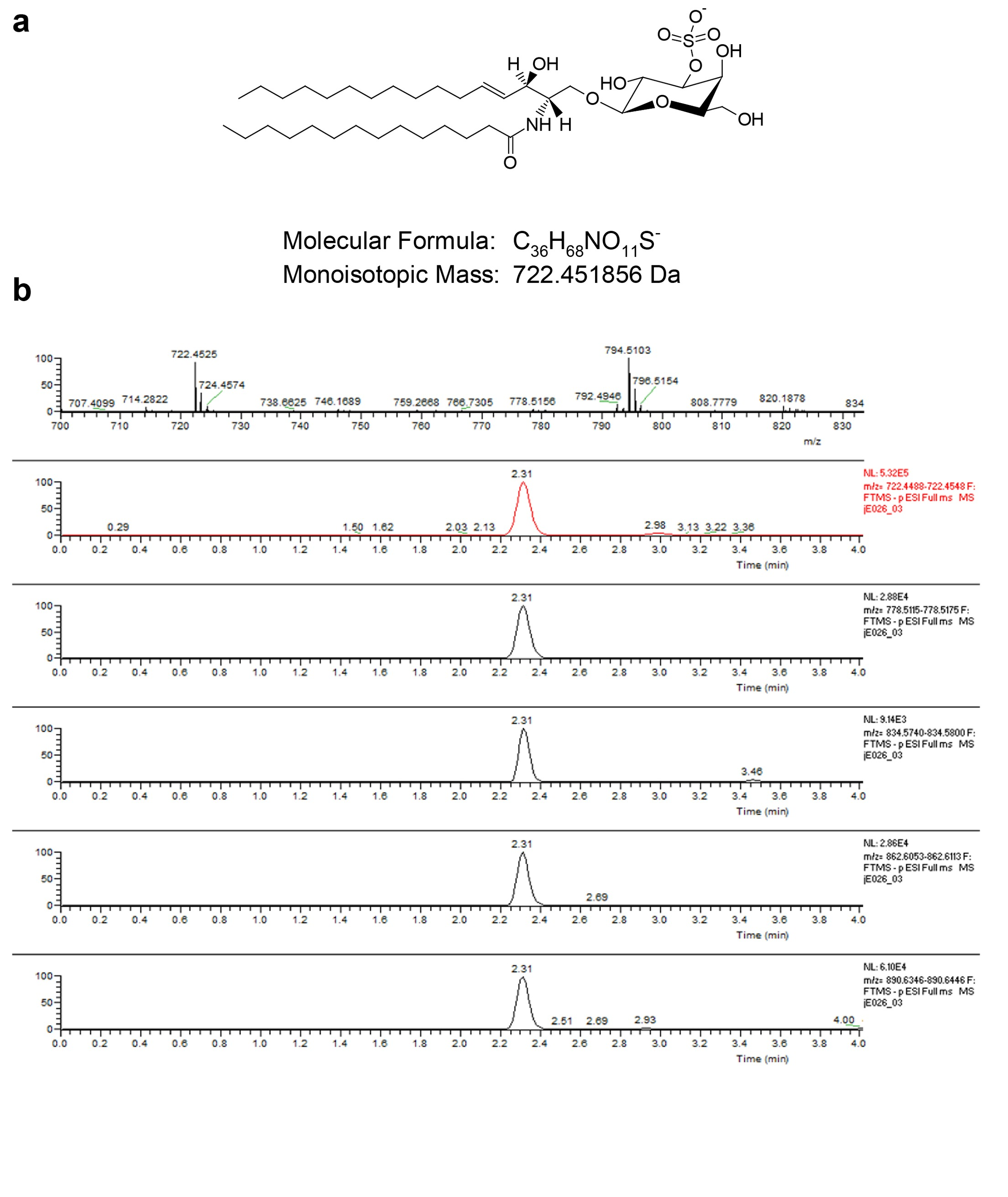

Supplement: S2 Fig — (a) Structure, molecular formula, and monoisotopic mass of sulfatide 30:1;2, which was used as an internal standard for the mass spectrometry analysis. (b) Negative ionization of the standard SHexCer 30:1;2. Below, further identification of the peak and retention time corresponding to its m/z = 722.4519. (TIF) [file pone.0194414.s002.tif]
